# Lipidomics Based on HILIC-ESI-HRMS Reveal Subtle Changes in the Mitochondrial Phospholipidome of Mouse Embryonic Fibroblasts Expressing Pathogenic Variants of Human OPA1 Protein

**DOI:** 10.3390/molecules31152719

**Published:** 2026-08-05

**Authors:** Andrea Castellaneta, Ilario Losito, Vito Porcelli, Serena Barile, Alessandra Maresca, Valentina Del Dotto, Penelope Magnoni, Claudia Zanna, Ludovica Sofia Guadalupi, Valeria Cinquepalmi, Cosima Damiana Calvano, David C. Chan, Valerio Carelli, Luigi Palmieri, Tommaso R. I. Cataldi

**Affiliations:** 1 Dipartimento di Chimica, Università degli Studi di Bari Aldo Moro, Via Orabona 4, 70126 Bari, Italy; andrea.castellaneta@uniba.it (A.C.); ludovica.guadalupi@uniba.it (L.S.G.); valeria.cinquepalmi@uniba.it (V.C.); cosimadamiana.calvano@uniba.it (C.D.C.); tommaso.cataldi@uniba.it (T.R.I.C.); 2 Centro Interdipartimentale di Spettrometria di Massa per Ricerche Tecnologiche (SMART), Università degli Studi di Bari Aldo Moro, Via Orabona 4, 70126 Bari, Italy; 3 Dipartimento di Bioscienze, Biotecnologie e Ambiente, Università degli Studi di Bari Aldo Moro, Via Orabona 4, 70126 Bari, Italy; vito.porcelli@uniba.it (V.P.); serena.barile@uniba.it (S.B.); luigi.palmieri@uniba.it (L.P.); 4 Programma di Neurogenetica, IRCCS Istituto delle Scienze Neurologiche di Bologna, Via Altura 3, 40139 Bologna, Italy; alessandra.maresca@isnb.it (A.M.); penelope.magnoni@ausl.bologna.it (P.M.); valerio.carelli@unibo.it (V.C.); 5 Dipartimento di Scienze Biomediche e Neuromotorie, Università degli Studi di Bologna, Via Altura 3, 40139 Bologna, Italy; valentina.deldotto2@unibo.it (V.D.D.); claudia.zanna@unibo.it (C.Z.); 6 Division of Biology and Biological Engineering, California Institute of Technology, 1200 East California Boulevard, Pasadena, CA 91125, USA; dchan@caltech.edu; 7 Consiglio Nazionale delle Ricerche (CNR)-Istituto di Biomembrane, Bioenergetica e Biotecnologie Molecolari, Via Giovanni Amendola, 122/O, 70126 Bari, Italy

**Keywords:** mitochondria, human OPA1, OPA1 variants, mouse embryonic fibroblasts, lipidomics, HILIC-ESI-HRMS

## Abstract

Pathogenic variants of the mammalian optic atrophy 1 (OPA1) protein, a key regulator of mitochondrial fusion, may lead to severe mitochondrial dysfunction and morphological alterations, and have also been proposed to be involved in remodeling the mitochondrial membranes lipid profile. To address this last issue, a lipidomic workflow based on HILIC-ESI-HRMS was applied to profile major classes of mitochondrial membrane phospholipids (PL), namely phosphatidylcholines (PCs), -ethanolamines (PEs), and -inositols (PIs), along with cardiolipins (CLs), in mouse embryonic fibroblasts knocked out for Opa1 gene (Opa1^−/−^ MEFs) and expressing human OPA1 isoform 1 (ISO1) or one of four well-known pathogenic variants (I382M, D603H, G439V and R445H). A total of 122 common sum compositions were recognized for PLs in the four classes across the five sample types. Chemometrics on the respective quantitative data indicated a lower prevalence of alk(en)yl/acyl species (O-PCs and O-PEs) within the PC and PE classes, along with a higher relative contribution of highly unsaturated PI and CL species, when severely pathogenic R445H and G439V variants were expressed. In contrast, the I382M variant was associated with a greater relative contribution of less-unsaturated PI and CL species. These findings indicate that pathogenic OPA1 variants are associated with distinct mitochondrial phospholipid profiles, opening interesting perspectives for future direct experimental validation of an eventual relationship existing between OPA1 variants, inter-organelle phospholipid trafficking, and mitochondrial dysfunction.

## 1. Introduction

OPA1 and Mitofusins (MFN1/MFN2) are GTPases that mediate mitochondrial membrane dynamics, regulating inner membrane (IMM) and outer membrane (OMM) fusion, respectively [1]. Pathogenic variants in OPA1 cause inner membrane fusion defects, resulting in mitochondrial fragmentation, cristae abnormalities, and oxidative phosphorylation deficiency, leading to isolated or syndromic dominant optic atrophy (DOA and DOA plus, respectively) [2]. Similarly, MFN2 pathogenic variants also disrupt mitochondrial dynamics, causing mitochondrial fragmentation and contributing to a spectrum of pathologies ranging from DOA plus to Charcot–Marie–Tooth type 2A (CMT2A) disease [3].

Phospholipids (PLs) represent the primary structural components of mitochondrial membranes, with phosphatidylcholines (PCs) and phosphatidylethanolamines (PEs) being the major classes, playing key roles in both IMM and OMM curvature, fluidity, and stability [4,5,6,7]. Notably, the IMM PL profile is crucial for several mitochondrial functions, including cristae formation, apoptosis, assembly of OXPHOS enzymes into supramolecular supercomplexes, and for mitochondrial morphology [8]. Other PL classes, namely phosphatidic acids (PAs) and cardiolipins (CLs), with the latter primarily located in the IMM, have been associated with the functionality of mitochondrial dynamics proteins [9,10,11,12,13]. Mitochondrial phospholipid biogenesis and metabolism are strictly related to areas of close membrane juxtaposition with other intracellular organelles, known as mitochondria-associated membranes (MAMs) [14]. In particular, the interaction with the endoplasmic reticulum (ER) plays a crucial role in the transfer of PLs in and out of mitochondria [15]. Recent findings by Lee et al. [16] highlighted the connection between organelle biogenesis (including peroxisomes, Golgi apparatus, and ER) and mitochondrial lipid composition. Disruptions in organelles’ function were shown to affect mitochondrial dynamics by altering lipid composition.

Based on this background, a potential relationship between the integrity of mitochondrial fusion proteins and mitochondrial phospholipid composition was hypothesized and subsequently supported by MS-based lipidomic studies. In particular, shotgun lipidomics revealed the occurrence of PC, lyso-PC and sphingomyelin (SM) remodeling in OPA1-deficient rat primary cortical neurons [17] and OPA1-deleted mouse embryonic fibroblasts (MEFs) [18]. Using the same approach, changes in the distribution of major PL classes and in the CL profile were evidenced in MEFs with OPA1 partially depleted via clustered regularly interspaced short palindromic repeats/associated protein 9 (CRISPR/Cas9) technology [19]. Notably, reversed phase liquid chromatography (RPLC) coupled with high-resolution MS revealed significant changes in the abundance of numerous lipid species across different classes in OPA1-disrupted MEFs [19]. In a recent investigation performed in our laboratory [20], PL remodeling occurring in mitochondria isolated from MEFs depleted of OPA1 or MFN1/MFN2 proteins was evaluated employing hydrophilic interaction liquid chromatography combined with high-resolution mass spectrometry using electrospray ionization (HILIC-ESI-HRMS). HILIC confirmed its renown potential in achieving a class-specific separation of PLs [21], enabling distinct profiling of major mitochondrial PL classes, namely PCs, PEs, PIs, and CLs, in conjunction with ESI-HRMS detection. The subsequent processing of PL quantitative data based on chemometrics emphasized the occurrence of a relevant PL remodeling upon depletion of fusion proteins, with a significant decrease in O-PCs and O-PEs compared to their diacylic counterparts, along with a general increase in side chain unsaturation across all PL classes [20].

In order to obtain an insight at a more subtle level on the potential involvement of OPA1 in mitochondrial PLs remodeling, major PL classes occurring in mitochondria of MEFs knocked out for Opa1 gene and then engineered to express either the wild-type human OPA1 isoform 1 (ISO1) or one of its four pathogenic variants, namely I382M, D603H, G439V, and R445H [22], were profiled in the present study using HILIC-ESI-HRMS. Among those variants, G439V and R445H are particularly relevant in pathological terms, being associated with severe forms of DOA plus, leading to extensive mitochondrial network fragmentation and mtDNA depletion/deletion [22]. In contrast, the I382M and D603H variants exhibit milder functional consequences [22], providing useful comparative models for understanding the spectrum of OPA1 pathogenic variants effects on mitochondrial lipid metabolism.

As discussed in detail in the present paper, the processing of quantitative HILIC-ESI-HRMS data obtained for mitochondrial PLs in the five cell lines, supported by chemometrics, provided insights into the subtle phospholipid remodeling associated with OPA1 variants, especially those already known to be more severely pathogenic. Although not directly associated in this study with specific ultrastructural or functional data, these insights might contribute to the future understanding of the complex link existing between the occurrence of OPA1 variants and mitochondrial dysfunction.

## 2. Results

### 2.1. Assessment of Mitochondrial Purity

**Figure 1:**
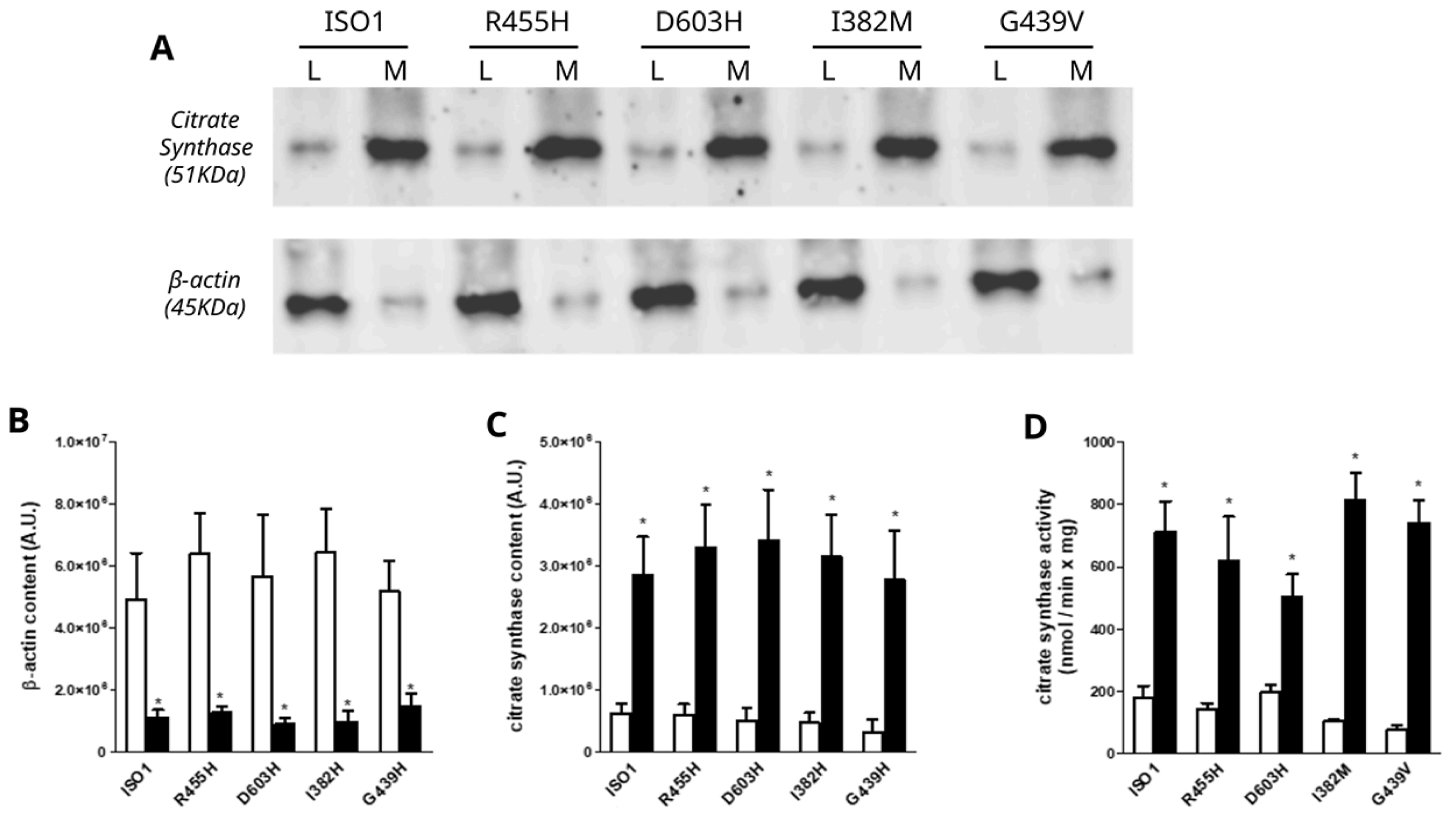
Mitochondrial samples purity check. (**A**) Representative Western blot analysis of three independent experiments carried out on cellular lysates and purified mitochondria relative to MEFs expressing either human OPA1 isoform 1 or one of its four pathogenic variants. (**B**,**C**) β-actin and C.S. contents (average ± SD) obtained by densitometric analysis of three independent experiments of Western blot. Significant differences in protein contents of lysates (white bars) and mitochondria (black bars) for each cell line are marked with * (*p* < 0.01, *t*-Student test). (**D**) C.S. activity, expressed as initial rate, measured in cell lysates and relative mitochondria, represented by white and black bars, respectively. Average and SD of three independent experiments are reported. Statistically significant differences in activity are marked with * (*p* < 0.01).

The purity and the enrichment of mitochondria isolated from MEF cells were assessed according to the procedure reported in Ref. [20]. Specifically, Western blot analysis was performed in triplicate to quantify cytosolic (β-actin) and mitochondrial (citrate synthase, C.S.) marker proteins. A representative Western blot of the cellular lysate and purified mitochondria from the five MEF lines under investigation is reported in Figure 1A (L = cellular lysates, M = mitochondrial fraction), indicating that the mitochondrial fraction (M) derived from each of the five cell lines was significantly enriched in citrate synthase (*p* < 0.01, as assessed by *t*-test) compared to the corresponding cellular lysate (L). This was confirmed by densitometric analysis of bands detected in three different experiments (Figure 1C). Conversely, β-actin was significantly more abundant in the lysates than in the corresponding mitochondrial fraction for each cell line (*p* < 0.01, as assessed by *t*-test), as evidenced in Figure 1B. Notably, the results obtained for β-actin in the case of mitochondria support the mitochondrial localization of a pool of this protein previously described by Xie and colleagues [23]. The activity of citrate synthase, an enzyme of the TCA cycle, was also measured and expressed as initial rate. As shown in Figure 1D, its specific activity was significantly increased (*p* < 0.01, *t*-test) upon mitochondrial purification in all the five cell lines, confirming Western blot results.

### 2.2. HILIC-ESI(−)-HRMS Analysis of MEF Mitochondrial Lipid Extracts

**Figure 2:**
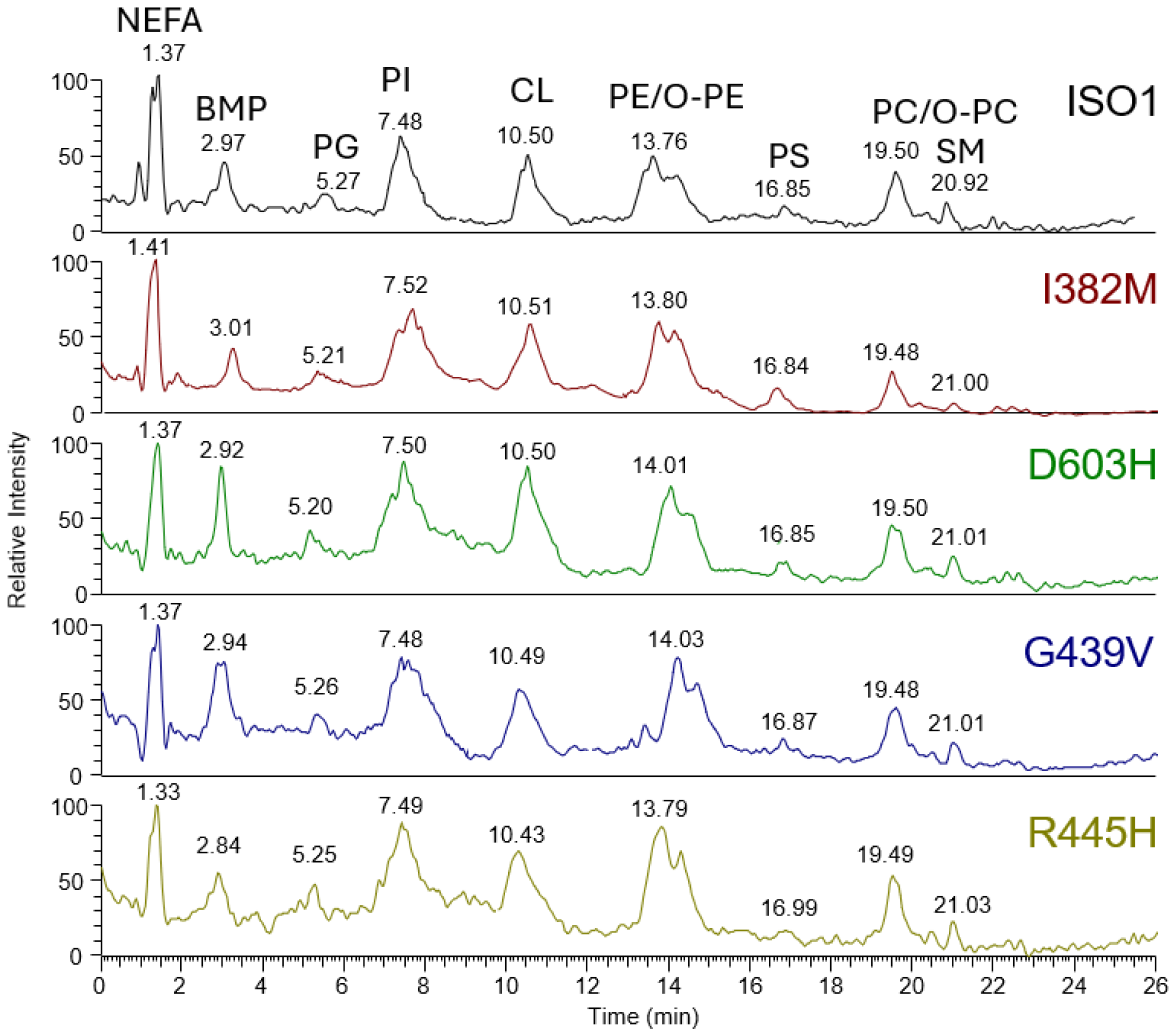
Comparison between total ion current (TIC) chromatograms resulting from HILIC-ESI(−)-HRMS analyses of mitochondrial lipid extracts obtained for Opa1^−^^/−^ MEFs expressing either human OPA1 isoform 1 (ISO1) or four of its pathogenic variants. The chromatograms show the separation of the following lipid classes: non-esterified fatty acids (NEFA), bis-monoacyl-glycerophosphates (BMP), phosphatidylglycerols (PG), phosphatidylinositols (PI), cardiolipins (CL), phosphatidylethanolamines (PE), phosphatidylserines (PS), phosphatidylcholines (PC) and sphingomyelins (SM). O-PE and O-PC indicate alk(en)yl/acyl PE and PC, respectively.

Figure 2 displays an example of total ion current (TIC) chromatograms obtained by HILIC-ESI(−)-HRMS for lipid extracts of mitochondria isolated from Opa1^−/−^ MEFs expressing either human OPA1 isoform 1 (ISO1) or one of the four pathogenic variants under study. PL classes were eluted from the HILIC column according to the following order: phosphatidylglycerols (PG), PI, CL, PE and O-PE, phosphatidylserines (PS), PC and O-PC, and finally sphingomyelins (SM). As expected, more hydrophobic PLs represented by bis-monoacyl-glycerophosphates (BMPs) and non-esterified fatty acids (NEFAs) were eluted earlier than the other lipid classes. While negative ionization efficiently detected most of the latter, PCs were most effectively ionized in positive ion mode due to the stable positive charge of the choline headgroup. Consequently, PCs were quantified using HILIC-ESI(+)-HRMS data.

The TIC traces indicate generally similar lipid class profiles for the five sample types, suggesting that species of PG, PS, and SM classes were present as minor constituents. This observation aligns with previous data from wild-type, Opa1^−/−^, and Mfn1/2^−/−^ MEF mitochondria [20], as well as from rat liver mitochondria [24]. The subsequent data processing was focused on major PL classes, i.e., PC, PE, PI and CL. For each class, HRMS spectra were averaged over the retention time intervals corresponding to their chromatographic bands. Representative spectra for PCs and PEs (Figure S1), and for PIs and CLs (Figure S2), referred to the lipid extracts of mitochondria isolated from Opa1^−/−^ MEFs expressing human OPA1 isoform 1 (ISO1), are provided in the Supplementary Information.

Accurate *m*/*z* ratios obtained from HRMS spectra clarified that both diacyl and alk(en)yl/acyl species were included in PC and PE classes (Figure S1 in Supplementary Materials). Their sum compositions ranged from 30 to 40 carbon atoms in both classes, with several species showing multiple double bonds (ranging from 5 to 8 total C=C). As for PIs and CLs, the sum compositions ranged from 34 to 40 and from 64 to 72, respectively (Figure S2 in Supplementary Materials), in accordance with the prevailing occurrence of fatty acyl chains including 16, 18 and 20 carbon atoms, as previously observed in NEFAs extracted from MEF mitochondria [20].

HRMS spectra also revealed the occurrence of a significant isotopic interference within each PL class, largely due to the close elution of similar compounds. In many cases, the M+0 isotopologue of one PL overlapped with the M + 2 isotopologue of another species from the same class with one fewer double bond, thus determining a Type-II isotopic interference. As discussed in our previous study [20], this overlap can distort both signal intensity and apparent *m*/*z* values, complicating accurate compound identification and quantification. Indeed, in the absence of Type-II isotopic interference correction, the occurrence of an abundant PL in a specific sample would lead to an overestimate of the abundance of a PL from the same class but including one C=C bond less along its side chains. To address this issue, the PL-specific spectra were first corrected for Type-II interference using the LIPIC software (version 1.0.0) [25]. Subsequently, this software was employed to calculate the total intensity of the full isotopic pattern for each PL from the corrected M+0 peak (Type-I correction), once its molecular formula was established.

**Figure 3:**
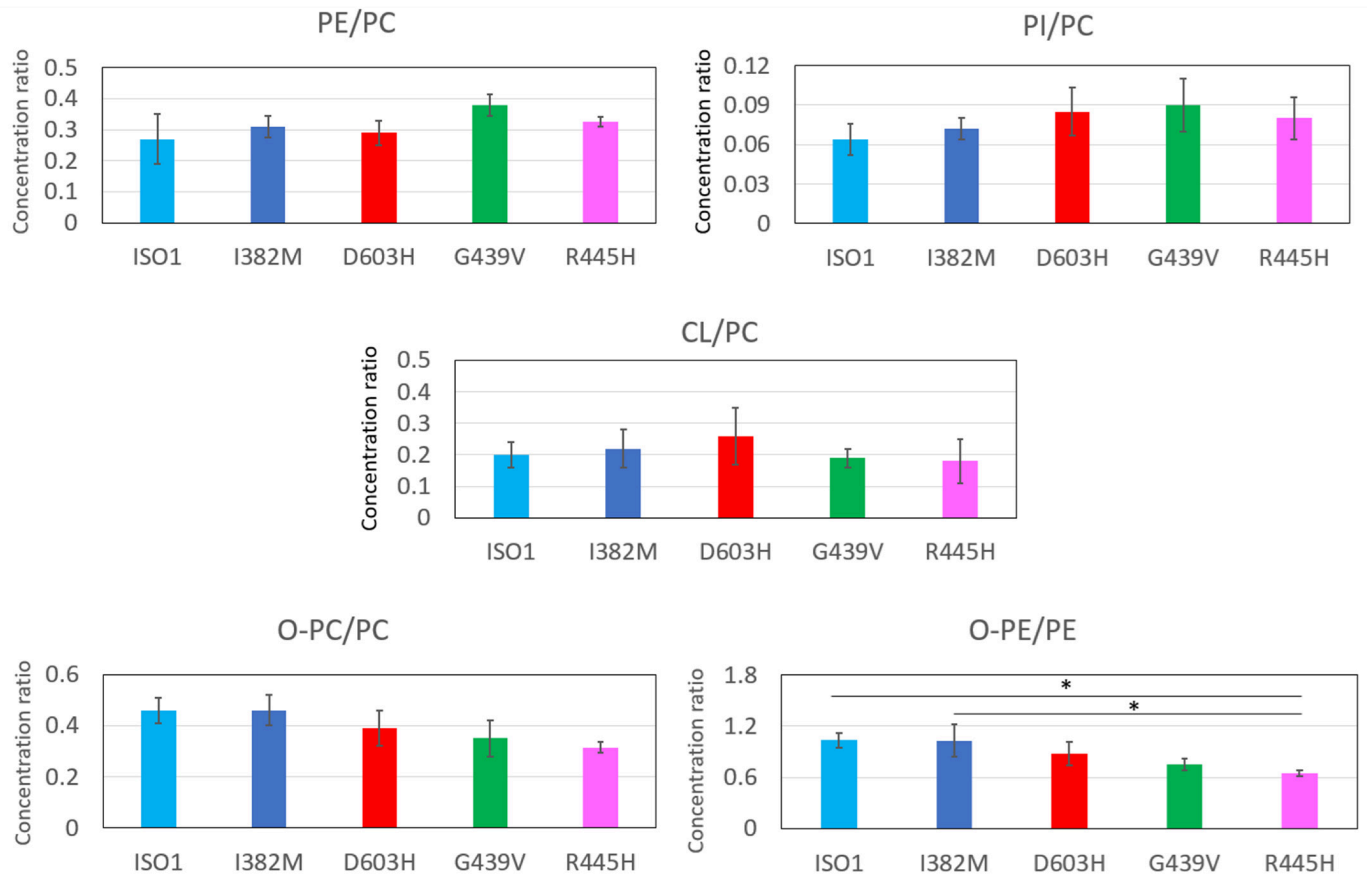
Concentration ratios observed between major PL classes detected in mitochondrial lipid extracts of Opa1^−^^/−^ MEFs expressing human OPA1 (ISO1) or four of its pathogenic variants. Ratios of PEs, PIs, and CLs to PCs are shown, with PCs selected as the reference class. Additionally, the ratios between alk(en)yl/acyl phosphatidylcholines (O-PCs) or phosphatidylethanolamines (O-PEs) and their respective diacylic counterparts are presented. All data are reported as average values, with error bars representing standard deviations, referring to three independent biological replicates for each cell line. Asterisks indicate significantly different ratios (*p* < 0.05).

The total signal intensity for each PL in a class was then employed to reconstruct a within-class abundance profile and estimate its concentration, by comparing its response to that of the internal standard for the same class. As described before, internal standards were added to each lipid extract immediately before HILIC-ESI-HRMS analysis. Our previous study [20] demonstrated that species within the same PL class exhibited similar ionization yields under the adopted HILIC conditions, which is a key requirement for the described quantitative approach. Concentrations of PCs, PEs, PIs, and CLs corresponding to specific sum compositions (which may represent several regioisomers or positional isomers), were used to estimate the total concentration of each PL class in each lipid extract. Concentration ratios were then calculated relative to the PC class, chosen as a reference due to its high abundance. Figure 3 reports their average values and standard deviations, determined from the three independent biological replicates available for each cell line. No statistically significant differences were inferred between ISO1 mitochondria and those harboring human OPA1 variants, in line with previous findings from wild-type, Opa1^−/−^, and Mfn1/2^−/−^ mitochondria [20]. Notably, the CL/PC ratios ranged from 0.18 to 0.26, thus being substantially higher than the ratios inferred from studies on the rat liver endoplasmic reticulum (0.017) [24] and nuclear membrane (0.023) [26]. This result strengthens data previously provided on the purity of mitochondrial preparations obtained during the present study. O-PC/PC and O-PE/PE concentration ratios were also determined for the five types of mitochondrial samples, and their average values (with standard deviations, *n* = 3) are also shown in Figure 3. Both ratios were almost identical in the case of mitochondria including wild-type human OPA1 (ISO1) and the I382M variant. On the other hand, both of them showed a decreasing trend when considering mitochondria including variants D603H, G439V and R445H and, as emphasized in the figure, the O-PE/PE ratio became also statistically lower (*p* < 0.05) than those found in ISO1 and I382M samples when the R445H variant, i.e., the one known to determine the most severe pathological consequences, was considered. Notably, the *p* value related to the decrease in the O-PC/PC ratio occurring for that variant compared to the ISO1 samples was just slightly greater (*p* = 0.066) than the adopted significance coefficient (0.05).

As discussed in Section 2.3, eventual further subtle variations occurring in PL class profiles associated with human OPA1 variants were subsequently investigated using the percentual abundances of sum compositions recognized for the major PL classes (illustrated for PIs and CLs in Figure S3 of Supplementary Materials) as input data for chemometrics.

### 2.3. Evaluation of Intra-Class PL Profiles by Chemometrics

**Figure 4:**
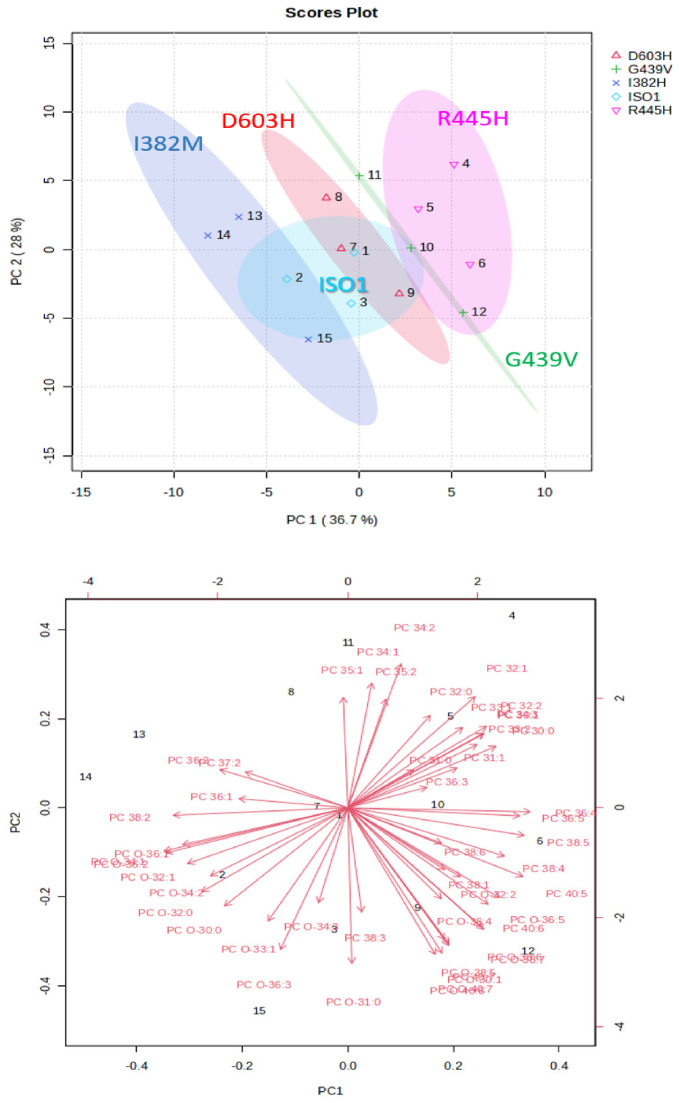
PCA score plot and biplot referred to the first two principal components based on intra-class abundances of PCs and O-PCs extracted from mitochondria of Opa1^−^^/−^ MEFs expressing human OPA1 isoform 1 (ISO1) or one of four pathogenic variants. 95% confidence regions for each sample type are also reported in the score plot. Samples are indicated only by the respective numbers in the biplot. Autoscaled data were used.

**Figure 5:**
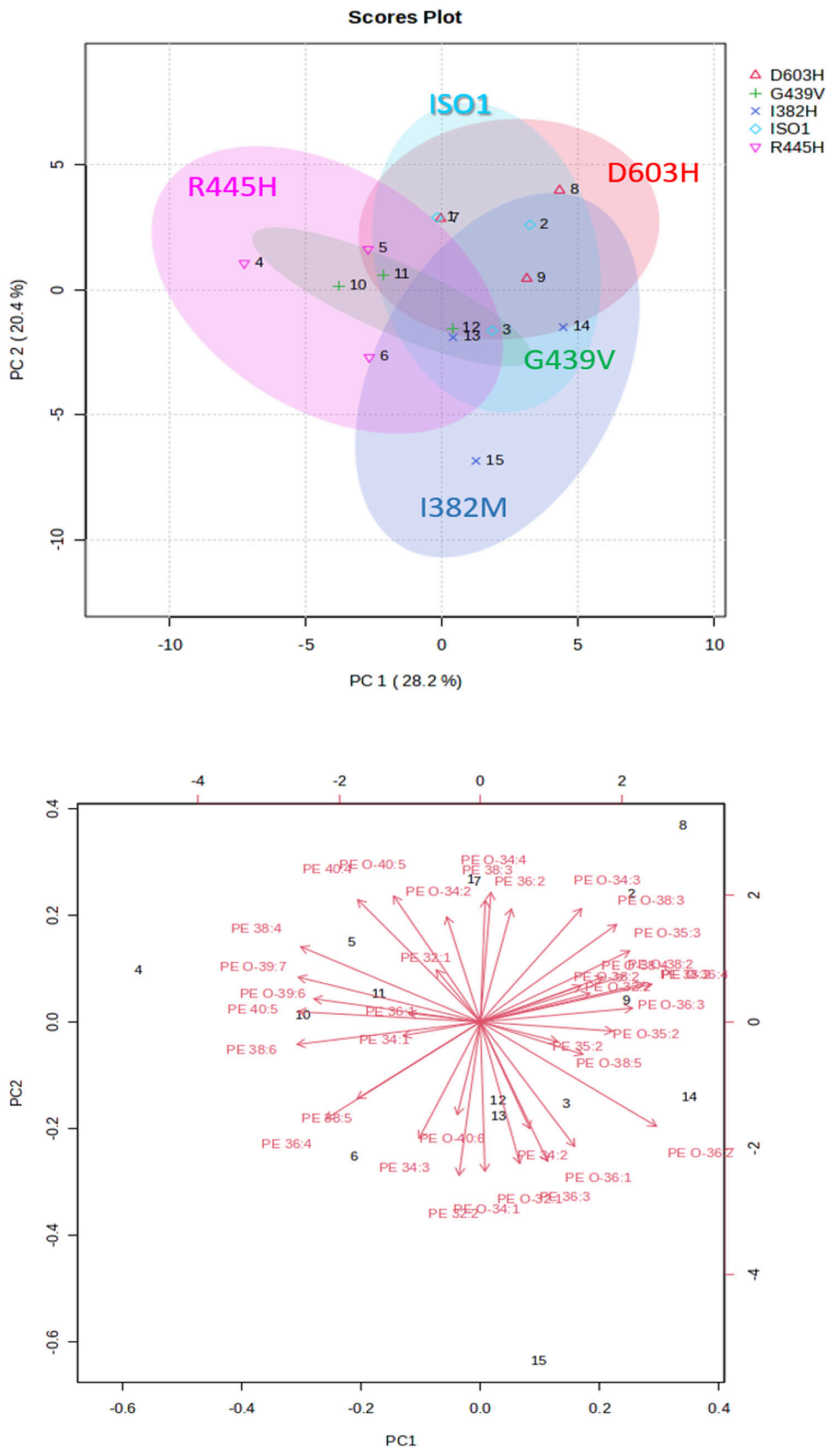
PCA score plot and biplot referred to the first two principal components based on intra-class abundances of PEs and O-PEs extracted from mitochondria of Opa1^−^^/−^ MEFs expressing human OPA1 isoform 1 (ISO1) or one of four pathogenic variants. 95% confidence regions for each sample type are also reported in the score plot. Samples are indicated only by the respective numbers in the biplot. Autoscaled data were used.

Intra-class relative abundances for 49 different PC/O-PC and for 38 distinct PE/O-PE sum compositions were measured across the 15 mitochondrial samples under study (three biological replicates per cell line). Following autoscaling, these values were used as input for Principal Component Analysis (PCA) performed separately for each PL class. The score plot for the first two principal components in the case of PCs, accounting for 64.7% of the total variance, is shown in Figure 4. In this plot, the colored areas, representing the 95% confidence regions estimated for each of the five sample groups, largely overlapped, indicating that overall PC profiles did not distinctly separate the sample types. The overlap was even more pronounced in the PCA score plot for PEs (Figure 5), where the first two principal components explained only 48.6% of the total variance. Based on these outcomes, the profiles of sum compositions for PC and PE classes would not be suitable to predict which specific group one sample belongs to, yet this was not the PCA goal in the present case. Indeed, PCA was rather performed to emphasize eventual trends in PL classes profiles related to OPA1 variants. From this point of view, a careful examination of the biplots corresponding to PCA score plots, also reported in Figure 4 and Figure 5, revealed a consistent trend, aligned with O-PL/PL ratios previously shown for PC and PE classes. Specifically, samples associated with the R445H variant (samples 4, 5 and 6) showed a higher relative contribution from diacyl species in both the PC and PE classes, suggesting a lower relative abundance of O-PL species compared to the other groups.

**Figure 6:**
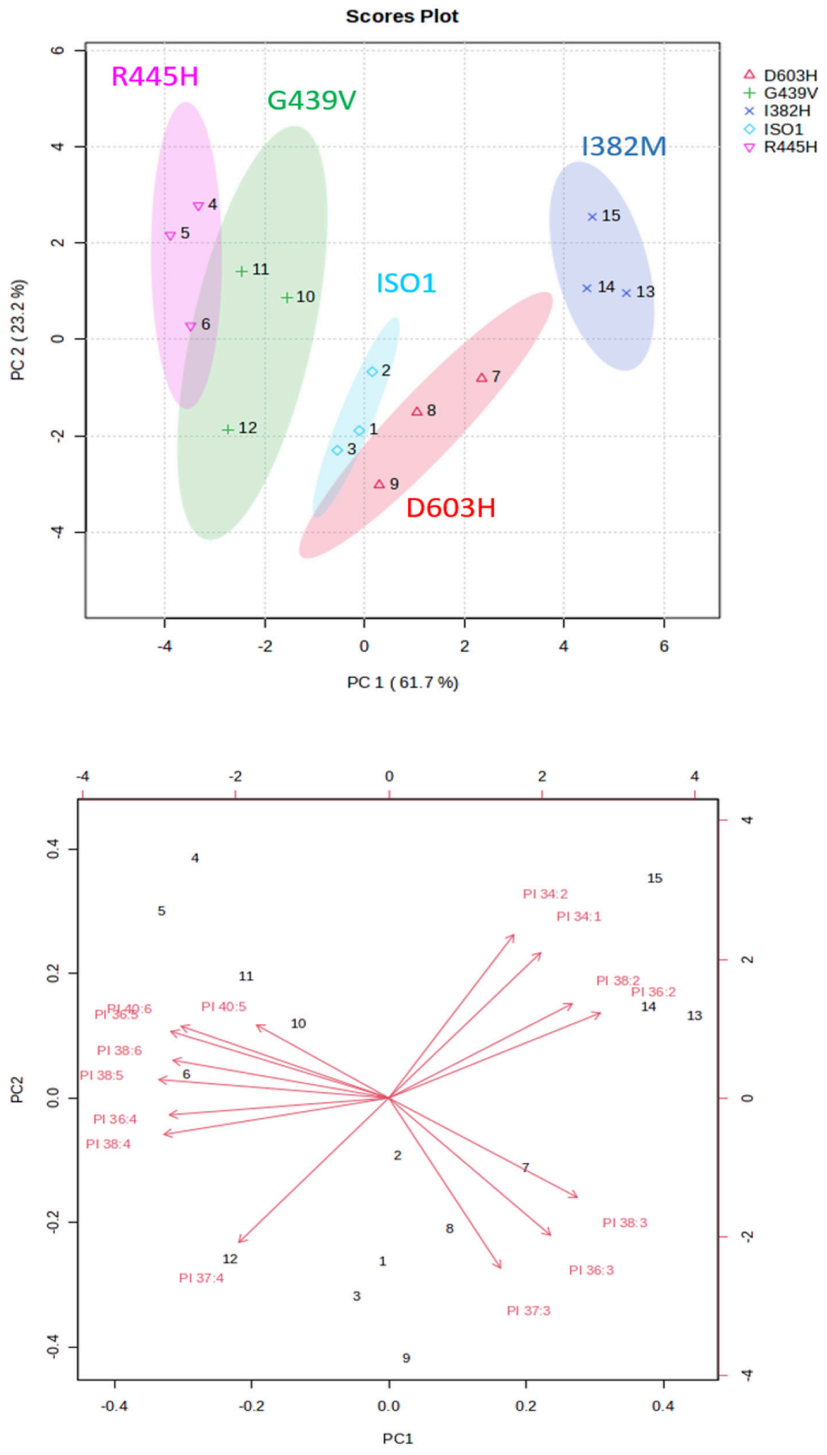
PCA score plot and biplot referred to the first two principal components based on intra-class abundances of PIs extracted from mitochondria of Opa1^−^^/−^ MEFs expressing human OPA1 isoform 1 (ISO1) or one of four pathogenic variants. 95% confidence regions for each sample type are also reported in the score plot. Samples are indicated only by the respective numbers in the biplot. Autoscaled data were used.

Notably, the relative abundance data obtained for the 15 PI sum compositions provided a much clearer separation at least for some sub-groups of samples, compared to PCs and PEs. As evidenced in Figure 6, MEF mitochondria expressing the I382M variant of human OPA1 were distinctly separated from the others in the PCA score plot, where the first two principal components accounted for 84.9% of total variance. Additionally, the 95% confidence regions for the ISO1/D603H and R445H/G439V couples of sample groups revealed two distinct sub-clusters, with minimal overlap (Figure 6). A detailed inspection of the biplot in Figure 6 reveals interesting trends related to fatty acyl chain unsaturation for PIs. Specifically, PIs including a lower number of carbon–carbon double bonds (1 to 3 C=C) were more relevant in mitochondria expressing the I382M mutation, while mitochondria carrying the highly pathogenic R445H and G439V variants exhibited a higher incidence of PIs with 4 to 6 C=C bonds. Samples belonging to ISO1 and D603H groups occupied intermediate positions in the biplot, reflecting a more balanced unsaturation profile for PIs.

These findings were further supported by Hierarchical Cluster Analysis (HCA) with heatmap visualization, as shown in Figure S4 in Supplementary Materials. Indeed, the three I382M samples clustered together in the HCA dendrogram, with the heatmap highlighting the prevalence of low-unsaturation PIs. Conversely, the R445H and G439V samples formed a separate sub-cluster characterized by a higher abundance of PIs with 4 to 6 C=C bonds. An exception was observed for G439V sample #12, which was grouped with the ISO1 and D603H samples due to a higher incidence of PIs with 3 C=C bond, likely driven by the unusually high level of PI 37:3 in that sample, as clearly shown in the heatmap. Overall, HCA confirmed the significant contribution of highly unsaturated PIs in mitochondria including R445H and G439V variants of OPA1.

**Figure 7:**
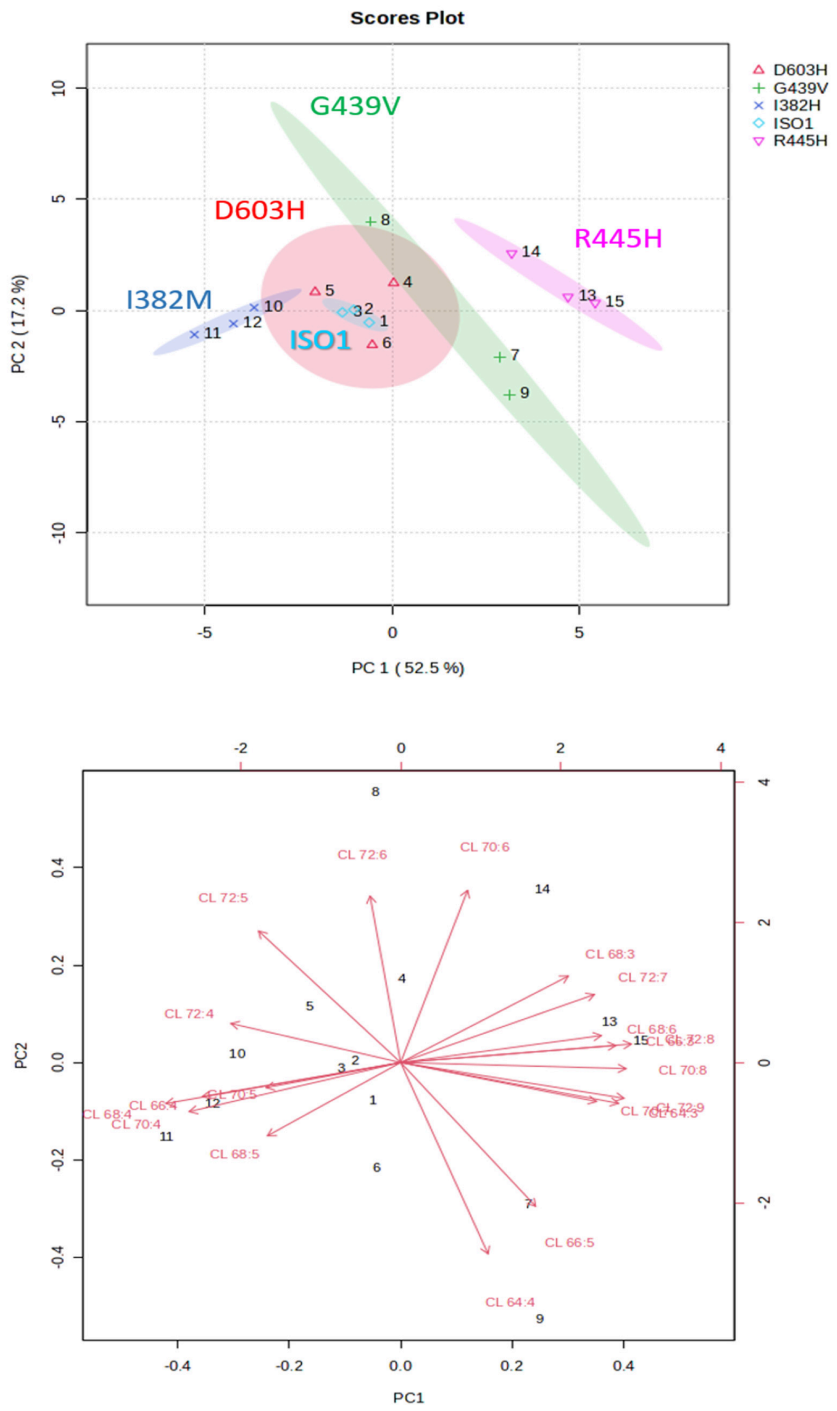
PCA score plot and biplot referred to the first two principal components based on intra-class abundances of CLs extracted from mitochondria of Opa1^−^^/−^ MEFs expressing human OPA1 isoform 1 (ISO1) or one of four pathogenic variants. 95% confidence regions for each sample type are also reported in the score plot. Samples are indicated only by the respective numbers in the biplot. Autoscaled data were used.

Chemometric analysis of the relative abundance data for the 20 common CL sum compositions across the five sample groups yielded intermediate results between those found for PCs/PEs and PIs. As illustrated in Figure 7, the PCA score plot based on the first two principal components, accounting for 69.7% of the total variance, clearly separated the R445H samples from the others. The corresponding biplot highlighted that highly unsaturated species (i.e., CLs with 6 to 9 C=C bonds) were more relevant among CLs in the R445H samples, while CLs with lower unsaturation levels (4 or 5 C=C bonds) were more abundant in the I382M samples. The remaining three sample types occupied intermediate positions in the plot. This outcome was further supported by HCA with heatmap visualization (Figure S5 in Supplementary Materials), even though G439V samples #7 and #9 clustered with R445H ones, in accordance with the proximity of the corresponding points to those related to R445H samples in the PCA score plot (see Figure 7).

## 3. Discussion

Based on the results obtained through HILIC-ESI-HRMS analyses, the expression in MEF mitochondria of OPA1 variants R445H and G439V determined the more relevant decrease in the alk(en)yl/acyl to diacyl species ratio for PCs and PEs. Notably, MEFs expressing the R445H and G439V variants were clearly distinguished from those expressing the I382M and D603H variants or ISO1 ones when considering the PCA processing of quantitative data on whole-cell PCs, SMs, lyso-PCs, amino acids and biogenic amines as variables [18]. These results are very relevant, considering that OPA1 variants R445H and G439V are closely associated with the severe DOA plus phenotype of dominant optic atrophy and to an extensive mitochondrial network fragmentation (nearly 100%) and a substantial reduction (~50%) in mitochondrial DNA copy number, as evidenced in a previous investigation performed by our research group on the same cell lines targeted in the present study [22]. In fact, bioenergetic analyses demonstrated that MEFs expressing the OPA1 R445H and G439V variants exhibited a marked reduction in intracellular ATP levels, comparable to those observed in Opa1^−/−^ cells, whereas the ATP content remained similar to that of WT cells in I382M- and D603H-expressing MEFs [22]. Seahorse analysis further revealed mutation-dependent respiratory defects, ranging from a mild reduction in the maximal/ATP-linked respiration ratio in I382M and D603H cells to a severe impairment in G439V and R445H ones, whose respiratory performance closely resembled that of Opa1^−/−^ MEFs [22]. Consistently with these findings, immunoblot analysis showed reduced steady-state levels of the OXPHOS subunits NDUFA9 (Complex I), UQCRC2 (Complex III), and COX IV (Complex IV) in G439V and R445H MEFs [22]. Blue Native PAGE combined with in-gel activity assays and densitometric analysis further demonstrated a reduction in the abundance of isolated Complex I as well as CI + III_2_, CI + III_2_ + IV, and higher-order CI + III_2_ + IV_n_ respiratory supercomplexes in these two mutant cell lines, whereas supramolecular organization was largely preserved in D603H and I382M MEFs. Finally, immunodetection of Complex V revealed a marked reduction of fully assembled ATP synthase (F_1_F_0_) together with the accumulation of lower-molecular-weight free F_1_ subcomplexes in G439V and R445H cells, similarly to Opa1^−/−^ MEFs, indicating partial ATP synthase disassembly [22].

In the same study, MEFs expressing the I382M variant were found to display a mitochondrial network comparable to that of ISO1 control cells. In contrast, the D603H mutation resulted in a significant increase in the proportion of cells with fragmented mitochondria and a corresponding decrease in cells exhibiting a filamentous network. As expected from their severe functional phenotype, MEFs harboring the G439V and R445H mutations showed an almost completely fragmented mitochondrial network [22]. Overall, these previously published findings establish the functional and bioenergetic phenotype of the same MEF lines investigated here and provide the biological context for the present lipidomic results. The present study does not include parallel measurements on mitochondrial membrane potential, ROS production, or respiratory activity. Consequently, although previously reported functional abnormalities in these cellular models provide relevant biological context, the current data do not establish a direct causal relationship between individual lipidomic changes and specific mitochondrial functional defects.

As highlighted in our previous lipidomic study concerning also MEFs not expressing WT OPA1 [20], the reduction in O-PCs and O-PEs observed when OPA1 is either not expressed or expressed with specific variants suggests that OPA1 may play an unexpected role in the biosynthetic pathway of these PLs. The latter involves the formation of 1-O-alkyl-sn-glycero-3-phosphates in peroxisomes, followed by their conversion into O-PCs and O-PEs within the ER and the subsequent transfer of the latter to mitochondria [27]. In our previous work [20], MEF mitochondria deprived of mitofusins exhibited a strong reduction in O-PC and O-PE levels. Since mitofusins play a key role in tethering mitochondria to the ER, facilitating lipid transport [28], this result was interpreted as being compatible with impaired transfer of these PLs from the ER to mitochondria. These observations provide a rationale for considering inter-organelle lipid trafficking as one possible interpretative context for the lower mitochondrial O-PL/PL ratios observed here. However, lipid transport was not measured in the present study, and the affected pathway cannot be identified from the lipidomic data alone.

The potential interplay between OPA1-dependent membrane organization, MICOS-associated contact-site architecture (with interactions reported with OPA1 at the IMM and with the two isoforms of Mitochondrial Rho GTPase, MIRO1/MIRO2, located in the OMM), mitochondrial dynamics, and phospholipid remodeling may provide a literature-based conceptual framework for interpreting the observed lipidomic phenotype [29,30,31]. This possibility should be regarded exclusively as a working hypothesis, because neither MICOS nor MIRO abundance, localization, interaction with OPA1, or functional status was investigated in the present study.

Recently, a significant reduction in mitochondrial O-PCs and O-PEs, compared to controls, was observed in the case of CAL51 cells (human breast carcinoma cells) subjected to the knock-out of the PEX26 gene [16]. The latter encodes the peroxisome assembly protein 26, a type II peroxisomal membrane protein that recruits Pex6-Pex1 complexes to peroxisomes, playing an important role in terms of protein import into them [32]. A similar reduction in mitochondrial O-PL/PL ratios was observed also when Golgi apparatus or ER biogenesis was disrupted [16]. The observed reduction in mitochondrial O-PL/PL ratios following disruptions in peroxisome and ER biogenesis supports the hypothesis that defects in lipid transfer between the ER, peroxisomes, and mitochondria, such as those potentially triggered by OPA1 pathogenic variants, can lead to similar outcomes. Notably, the same work emphasized that supplementation with ether-lipid precursors can effectively restore mitochondrial morphology and function in cells exhibiting biogenesis defects in peroxisomes, Golgi apparatus or ER [16].

However, these observations cannot be directly extrapolated to MEFs expressing human OPA1 pathogenic variants. Dedicated experiments and functional validations will be required to evaluate if ether-linked phospholipid supplementation can lead to recover normal O-PL/PL ratios in mitochondria obtained from these cells.

As previously discussed, the intra-class profiles of PIs and CLs were notably affected by the expression of some OPA1 variants, with differences occurring in the degree of unsaturation of the acyl chains of those phospholipids. Specifically, a higher incidence of highly unsaturated species was observed in the presence of the highly pathogenic R445H and G439V variants, whereas less-unsaturated PLs were associated with the I382M variant, with ISO1 and D603H samples exhibiting intermediate unsaturation profiles. A deeper evaluation of PCA biplots (Figure 3 and Figure 4) showed that these trends were confirmed also for PC and PE classes, although partially masked by variations involving their O-PL species. Notably, an enrichment in polyunsaturated PLs was previously observed also in our study on mitochondria lacking OPA1 or mitofusins [20]. The resulting increase in membrane fluidity may thus promote the morphological features systematically observed in mitochondria lacking fusion proteins, namely, a more rounded shape and a significant reduction in the number of cristae, as evidenced in several studies involving electron microscopy [19,33,34,35,36,37,38,39,40]. In one of these investigations, performed by our research group [37], the mitochondrial ultrastructure was investigated on Opa1-knockout MEFs reconstituted with human OPA1 ISO1 and, by comparison, with ISO1 carrying the pathogenic variant G300E, which is located in the same GTPase domain as the G439V and R445H variants. Notably, G300E, G439V and R445H mutations in OPA1 led to remarkably similar functional phenotypes, with G300E cells displaying severely impaired mitochondrial respiration, dramatically reduced proliferative capacity under oxidative conditions, marked respiratory supercomplex disorganization, and partial ATP synthase disassembly, resulting in an energetic phenotype largely overlapping that of Opa1-null cells [37]. When electron microscopy was applied to mitochondria including the G300E OPA1 variant, a more rounded shape generally emerged but, more importantly, a severe cristae disorganization was observed, with a maximum of one crista and a swollen, hypodense matrix being found in approximately 60% of cases, compared to less than 10% of cases when ISO1 was expressed [37]. The striking overlap of the functional phenotype observed for G300E, G439V, and R445H variants suggests that the last two mutations are likely to be associated with a degree of cristae disorganization comparable to the G300E one, even if this is an inference that needs to be confirmed experimentally in the future.

Interestingly, O-PL species, especially those including a vinyl-ether moiety (plasmalogens), have been reported to play a key role in the transition from a lamellar (no curvature) to an inverted hexagonal (negative curvature) phase in model membranes [41] and negative curvature is relevant to ensure the correct shape of cristae. The previously hypothesized alteration of O-PL transport towards mitochondria related to specific OPA1 variants might thus have important consequences also on the mitochondrial ultrastructure. Notably, Renne et al. [42] proposed that mitochondrial PL unsaturation in yeast is also influenced by lipid transport between the ER and mitochondria. Alterations of this process related to the presence of specific variants of OPA1 may thus be responsible for the general increase in side chain unsaturation observed in MEF mitochondrial PLs during the present study.

Being designed as an MS-based lipidomics investigation, this work did not include parallel ultrastructure imaging, along with new molecular or functional assays, on mitochondria including OPA1 variants. Accordingly, the phospholipid alterations reported here cannot be interpreted as direct evidence of changes in mitochondrial cristae architecture and membrane fluidity, as well as changes in mitochondrial membrane potential, ROS production or respiratory activity. Previously published characterizations of the same MEF lines, together with ultrastructural observations in related cell models, provide a relevant biological context but remain distinct from the lipidomic data reported here.

## 4. Materials and Methods

### 4.1. Experimental Design

Five lines of MEFs were generated to express either the native isoform 1 of human OPA1 or one of four pathogenic variants (I382M, D603H, G439V, and R445H) in MEFs previously knocked out for murine Opa1 gene (Opa1^−/−^) [22]. At this aim, plasmids (pMSCV-puro vector) expressing the human OPA1 isoform 1 and the isoform 1 bearing mutations G439V or R445H, previously described [12], were employed. Additionally, plasmids expressing the human OPA1 isoform 1 were mutagenized to generate mutations I382M and D603H, as described in Ref. [22]. OPA1^−/−^ MEFs were generated in the laboratory of Prof. David Chan [43] and kindly provided by him. Retrovirus production and transduction of OPA1^−/−^ MEFs were performed during this study as described previously [44]. Briefly, retroviral expression vectors were co-transfected with the ecotropic retroviral packaging vector pCLEco into HEK293T cells. Retroviral supernatants were collected 48 h after transfection and used to transduce OPA1^−^^/−^ MEFs. Transduced cells were subsequently selected by puromycin treatment to obtain a stable pooled population.

Each cell line was cultured in triplicate, thus providing three independent biological replicates. Following the protocol adopted in our recent study on Opa1^−/−^ MEFs [20], a mitochondrial pellet was obtained from each biological replicate and resuspended in water. Each suspension was then divided into three equal aliquots and phospholipids were extracted from each of them and subsequently identified at the level of sum composition and quantified using HILIC-ESI-HRMS. The concentrations obtained from the three extracts for each lipid species were finally averaged to yield a mean value per mitochondrial pellet, thereby accounting for extraction and analysis variability.

### 4.2. Chemicals

LC-MS grade water, methanol, and acetonitrile (used for HILIC mobile phase preparation), as well as HPLC-grade methanol, and chloroform (used for lipid extraction), and reagent-grade ammonium acetate (used as HILIC mobile phase additive) were purchased from Merck (Milan, Italy). The EQUISPLASH^®^ LIPIDOMIX^®^ quantitative mass spectrometry internal standard, consisting in a solution of 13 deuterated lipids at a concentration of 100 μg/mL each, was purchased from Avanti Polar Lipids (Alabaster, AL, USA). This standard includes 1-pentadecanoyl-2-oleoyl(d7)-sn-glycero-3-phosphocholine, 1-pentadecanoyl-2-oleoyl(d7)-sn-glycero-3-phosphoethanolamine, and 1-pentadecanoyl-2-oleoyl(d7)-sn-glycero-3-phosphoinositol, which were used as internal standards for the quantification of PCs, PEs and PIs, respectively. Additionally, standard 1′,3′-bis[1,2-dimyristoyl-sn-glycero-3-phospho]-glycerol (CL 14:0/14:0/14:0/14:0), purchased from Merck (Milan, Italy), was used as an internal standard for the quantification of CLs, after verifying that it was not detectable in MEF mitochondrial lipid extracts.

### 4.3. Cells, Culture Conditions and Mitochondria Isolation

MEFs expressing human OPA1 isoform 1 or one of its four pathogenic variants of interest were cultured at 37 °C in a humidified atmosphere including 5% CO_2_ and in a high glucose Dulbecco’s Modified Eagle’s Medium (DMEM, Euroclone, Milan, Italy) supplemented with 10% fetal bovine serum (Euroclone, Milan, Italy), 50 U of penicillin G/mL, 50 μg/mL of streptomycin sulfate, and 2 mM of L-glutamine (Merck, Milan, Italy). Cell counting was performed using the Scepter™ Automated Cell Counter (Sigma-Aldrich, Milan, Italy), following the manufacturer’s instructions. Trypan blue visualization was performed in parallel, in order to exclude significant differences in live or dead trypsinised cells ratio among the tested cells. Dounce homogenization was adopted for the isolation of mitochondria from MEF cell lines, using the protocol of the mitochondria isolation kit for cultured cells (product code 89874) purchased from Thermo Fisher Scientific (Rodano, Italy), slightly modified to obtain a more purified fraction of mitochondria, with more than 50% reduction in lysosomal and peroxisomal contaminants. According to a previous study [45], Dounce homogenization is expected to reduce nuclear contaminations compared to differential centrifugation. In the present case, the procedure was performed after incubating cells (20 × 10^6^) with 800 μL of the mitochondria isolation kit Reagent A for 2 min on ice, then 800 μL of Reagent C were added, and the suspension was centrifuged for 10 min at 700× *g* and 4 °C. The resulting supernatant was centrifuged for 15 min at 3000× *g* and 4 °C, then the mitochondrial pellet was retrieved and washed with 500 μL of Reagent C. Finally, centrifugation at 12,000× *g* at 4 °C was performed for 5 min, and the resulting pellet was collected. The BCA protein assay reagent (product code 23227, Thermo Fisher Scientific, Rodano, Italy) was adopted to evaluate the protein concentration related to mitochondria.

### 4.4. Western Blotting and Enzymatic Assays

The purity of mitochondrial samples was assessed by immunodetection of citrate synthase and β-actin in cellular lysates and purified mitochondria relative to MEFs expressing isoform 1 of human OPA1 or one of its four pathogenic variants. Cells were lysed in radioimmunoprecipitation assay (RIPA) buffer (product code 89900, Thermo Fisher Scientific, Rodano, Italy), and 20 μg of total proteins from whole cells or isolated mitochondria were treated with 10 mM Tris/HCl pH 6.8, 2% SDS, and 5% β-mercaptoethanol. Samples were then separated on two different 12% polyacrylamide gels and transferred onto nitrocellulose membranes. The first membrane was blotted using an antibody against the mitochondrial marker citrate synthase (C.S.) (MA5-17264, Invitrogen, Waltham, MA, USA), while the second was treated with the antibody against the cytosolic protein β-actin (MA5-15739, Invitrogen, Waltham, MA, USA). Both membranes were subsequently incubated with secondary antibodies host in mouse and rabbit, respectively, and conjugated with horseradish peroxidase (product code 31430, Thermo Fisher Scientific, Waltham, MA, USA). As loading control before Western blot assay, membranes were stained with red Ponceau S and then blotted for the indicated proteins. Quantification of β-actin and citrate synthase was performed by densitometric analysis of three independent Western blot experiments, using the Image Lab™ Touch software version 6.1 (Bio-Rad Laboratories, Hercules, CA, USA).

Additionally, cellular lysates and mitochondrial samples were subjected to citrate synthase activity assays using 30 μg of proteins for both and based on the measurement of the absorption at 412 nm related to thionitrobenzoic acid (TNB). In the presence of saturating concentrations of substrates acetyl-CoA, oxaloacetate, and dithionitrobenzoic acid (DNTB), the amount of TNB produced is directly proportional to the enzymatic activity of citrate synthase [46].

### 4.5. Lipid Extraction from MEF Mitochondria

Lipid extraction from mitochondria was performed by a slightly modified version of the Bligh and Dyer (BD) protocol [47] described in our recent paper [20]. Specifically, mitochondrial pellets obtained from each MEF cell line were first resuspended in LC-MS grade water at room temperature, then the suspension was divided into three equal 800 μL aliquots. Each aliquot underwent lipid extraction with a 2:1 (*v*/*v*) methanol/chloroform mixture. After centrifugation at 4500× *g* for 10 min, the lipid-rich supernatant was carefully collected. Equal volumes of water and chloroform were then added to the supernatant, followed by 1 min of vortexing. A second centrifugation at 4500× *g* for 10 min allowed the separation of the phases, and the lower chloroform-rich phase (1.5 mL), containing the lipids, was collected. This phase was evaporated under a nitrogen stream, and the resulting residue was redissolved in a 2:1 (*v*/*v*) methanol/chloroform mixture, which is more suitable for HILIC-ESI-HRMS analysis. The solution was rapidly transferred into a screwcap vial whose headspace was saturated with nitrogen to minimize lipid oxidation; the vial was then stored at −20 °C until analysis. Immediately before HILIC-ESI-HRMS analysis, an aliquot of the extract was spiked with appropriate volumes of the EQUISPLASH^®^ LIPIDOMIX^®^ solution (Avanti Polar Lipids, Alabaster, AL, USA) and a 100 μg/mL solution of the CL 56:0 standard, to ensure a final concentration of 3 μg/mL for each of the phospholipids adopted as internal standards for the four classes of interest.

### 4.6. HILIC-ESI-HRMS Instrumentation and Operating Conditions

An Ultimate 3000 HPLC quaternary chromatographic system coupled to a Q-Exactive high-resolution Fourier-transform quadrupole-Orbitrap mass spectrometer (Thermo Fisher, West Palm Beach, CA, USA) mounting a heated electrospray ionization (HESI) source, was adopted to perform HILIC-ESI-HRMS analyses of lipid extracts obtained from MEF mitochondria, based on the same settings described in our recent paper [20]. Chromatographic separations were performed using an Ascentis Express HILIC column (15 cm length, 2.1 mm internal diameter) packed with core–shell 2.7 μm silica particles (Supelco, Bellefonte, PA, USA). A guard column (2 cm length, 2.1 mm internal diameter) packed with the same type of particles was placed before the analytical column. Lipid separations were accomplished at a 0.3 mL/min flow rate, after injecting a 5 μL sample volume and through the adoption of a binary elution gradient based on an acetonitrile/water (97:3 *v*/*v*) mixture as solvent A and a methanol/water (90:10 *v*/*v*) mixture as solvent B. Both solvents contained ammonium acetate 2.5 mM, used as a ionization adjuvant. The elution program included the following steps: (0–10 min) linear increase in B from 2 to 20%; (10–15 min) linear increase in B from 20 to 50%; (15–20 min) isocratic at 50% B; (20–25 min) return to 2% B; (25–30 min) column reconditioning at 2% B.

The settings employed for the HESI interface and the ion optics of the Q-Exactive spectrometer were the following: (sheath gas flow rate) 35 a.u.; (auxiliary gas flow rate) 15 a.u.; (spray voltage) ±2.5 kV for positive/negative ion acquisitions; (capillary temperature) 320 °C; (S-lens RF level) 100. HRMS full scan acquisitions were obtained for each chromatographic run in a 100–2000 *m*/*z* interval operating the mass spectrometer at its maximum resolving power (140,000 at *m*/*z* 200). The Orbitrap fill time and the Automatic Gain Control (AGC) level for the spectrometer were set as 200 ms and 1 × 10^6^, respectively. As an additional acquisition, All Ion Fragmentation (AIF) measurements (alternated to full scan acquisitions) were performed during chromatographic runs by setting a systematic fragmentation of lipid ions in the higher-energy collisional dissociation (HCD) cell of the Q-Exactive spectrometer, operated at a 35% Normalized Collisional Energy (NCE). The mass spectra referred to lipid ion fragments were acquired in a 150–800 *m*/*z* interval, using the maximum available mass resolving power. AIF acquisitions were employed to recognize chromatographic bands related to specific PL classes through the extraction of currents related to class-specific product ions (*vide infra*). The mass spectrometer was calibrated on a daily base by infusing, at a 5 μL/min flow rate, calibration solutions for positive or negative polarity acquisitions provided by the instrument manufacturer. As a result, mass accuracy was always better than 5 ppm.

### 4.7. Processing of HILIC-ESI-HRMS Data

HILIC-ESI-HRMS data were processed following the same approach recently described in Ref. [20], with a focus on the four major PL classes detected in mitochondrial extracts, i.e., PC, PE, PI and CL. First, PL class-specific extracted ion current (XIC) chromatograms were obtained from HILIC-ESI-AIF-HRMS datasets through the extraction of ion currents for PL headgroups-related product ions, namely ions having exact *m*/*z* 184.0733 ([C_5_H_15_NO_4_P^+^]), 196.0380 ([C_5_H_11_NO_5_P^−^]), 241.0119 ion ([C_6_H_10_O_8_P^−^]) and 152.9958 ([C_3_H_6_O_5_P^−^]), for PC, PE, PI and CL, respectively.

PL class-specific chromatographic bands detected in each of the four extracted ion current (XIC) traces guided the subsequent averaging of high-resolution mass spectra from HILIC-ESI-HRMS datasets. The resulting spectra were then processed using the Alex^123^ eXtractor routine, included in the Alex^123^ software, freely available on the World Wide Web (www.mslipidomics.info) [48]. In particular, a comparison was made between *m*/*z* ratios for which signals were detected in HRMS spectra and theoretical values included in a Target List preliminarily generated using the Alex^123^ *Target List Generator* routine, referred to the M+0 isotopologues of singly charged ions for PCs, PEs, and PIs, and doubly charged ions for CLs. The constraint that a match was acceptable when the difference between the experimental and theoretical *m*/*z* values was lower than 0.05 units was set. As a result, lists of sum compositions of PLs occurring in each class were obtained, reported according to the notation proposed by Liebisch et al. [49], i.e., as C:D for diacylic PLs and O-C:D for alk(en)ylic/acylic PLs, with C and D representing, respectively, the total numbers of carbon atoms and double C=C bonds on the two side chains. The recognized PLs for each class were subsequently subjected to an intensity-based filtering, i.e., to the selection only of those for which the intensity of the M+0 isotopologue peak was higher than 1% of that of the base peak in the corresponding spectrum. Signal intensities for selected PLs, subjected to Type-I and Type-II isotopic corrections using the LIPIC software version 1.0.0 [25], were employed to estimate the relative abundances of each PL in its class. As proved experimentally in our recent study [20], this is a reliable approach, since the ionization yield for species belonging to the same PL class is comparable under HILIC conditions.

Relative abundance values resulting for each PL after the analysis of the three extraction replicates obtained from each mitochondrial pellet were averaged and the corresponding relative standard deviations (RSD) were calculated. Relative abundances for which an RSD higher than 20% was found were not included in chemometrics elaborations referring to all cell lines, so that only species with reproducible concentrations could be included in the latter. Notably, corrected peak intensities obtained for PL species after the analysis of mitochondrial lipid extracts preliminarily spiked with deuterated internal standards for PC, PE and PI and with CL 56:0 were used to estimate the concentrations of those species in each sample. Afterwards, total concentrations of PCs, PEs, PIs, and CLs in each sample were calculated and used to compare the profiles of major PL classes in the five MEF lines under consideration.

### 4.8. Statistical Analysis and Chemometrics

Average PL class concentration ratios obtained for the five types of mitochondrial samples under investigation were subjected to the HSD Tukey test to assess statistically significant differences (*p* = 0.05). The relative abundances referred to PCs, PEs, PIs, and CLs, obtained for each of the three biological replicates of the five MEF lines, according to the previously described procedure, were processed by Principal Component Analysis (PCA) and Hierarchical Cluster Analysis (HCA) with heatmap visualization, performed using MetaboAnalyst version 6.0 [50] (www.metaboanalyst.ca). Both PCA and HCA were based on autoscaled data. In the case of HCA, Euclidean distances and the average linkage agglomeration algorithm were employed. Score plots/biplots obtained from PCA and dendrograms with heatmaps generated after HCA provided visual insights into the data structure. In addition, 95% confidence ellipses were added to PCA score plots, as the samples were preliminarily classified according to the respective MEF line.

## 5. Conclusions

HILIC-ESI-HRMS enabled a detailed characterization of the mitochondrial phospholipidome of Opa1^−/−^ MEFs expressing human OPA1 isoform 1 or four of its pathogenic variants. The most pronounced differences in mitochondrial phospholipid profiles were observed in mitochondria expressing severely pathogenic OPA1 variants R445H and G439V. In these cases, lower ratios between alk(en)yl/acyl PCs and PEs and their diacyl counterparts and a greater relative contribution of highly unsaturated PI and CL species were observed, compared to mitochondria expressing OPA1 isoform 1. In contrast, the I382M variant was associated with a greater relative contribution of less-unsaturated PI and CL species. These findings suggest that pathogenic OPA1 variants are associated with distinct, although quantitatively subtle, changes in mitochondrial phospholipid composition. A possible relationship among OPA1-dependent membrane organization, inter-organelle phospholipid trafficking, and the observed lipidomic profiles may be considered as a working hypothesis. However, the present results do not establish the molecular mechanism underlying these alterations or a direct causal relationship with mitochondrial morphology or function. The phospholipid profiles characterized here provide a basis for future studies combining lipidomics with ultrastructural, molecular, and functional analyses.

## Data Availability

The original Western blot data presented in this study are openly available in the Zenodo repository. The data presented in this study are available on request from the corresponding author.

## Supplementary Materials

The following supporting information can be downloaded at: https://www.mdpi.com/article/10.3390/molecules31152719/s1, Figure S1: HRMS spectra averaged under chromatographic bands referred to PCs and O-PCs (upper panel), and PEs and O-PEs (lower panel), obtained after the HILIC-ESI-HRMS analysis, in positive and negative polarity, respectively, of the mitochondrial lipid extract referred to a sample of Opa1^−/−^ MEFs expressing isoform 1 of wild-type human OPA1 (ISO1). For the sake of clarity, only peak signals referred to the most abundant monoisotopic ions were labeled with the sum composition of the corresponding PL. In the case of alk(en)yl/acyl PL the sum composition label is reported as O-C:D, with C and D representing the total number of carbon atoms and C=C bonds of the side chains. Figure S2: HRMS spectra averaged under chromatographic bands referred to PIs (upper panel) and CLs (lower panel), obtained after the HILIC-ESI-HRMS analysis in negative polarity of the mitochondrial lipid extract referred to Opa1^−/−^ MEFs expressing isoform 1 of wild-type human OPA1 (ISO1). For the sake of clarity, only peak signals referred to the most abundant monoisotopic ions were labeled with the sum composition of the corresponding PL. The PLs sum composition label is reported as C:D, with C and D representing the total number of carbon atoms and C=C bonds of the side chains. Figure S3: Percentual abundance values related to specific sum compositions recognized for PI and CL classes obtained after the HILIC-ESI(−)-HRMS analysis of mitochondrial lipid extracts obtained from Opa1^−/−^ MEFs expressing isoform 1 of wild-type human OPA1 (ISO1) or four of its pathogenic variants (I382M, D603H, G439V, R445H). Average values obtained for three biological replicates and error bars representing standard deviations are reported. Figure S4: Dendrogram with heatmap obtained by Hierarchical Cluster Analysis (HCA) on autoscaled data concerning the intra-class relative abundances of PIs resulting from the HILIC-ESI(−)-HRMS analysis of mitochondrial lipid extracts obtained from Opa1^−/−^ MEFs expressing isoform 1 of wild-type human OPA1 (ISO1) or four of its pathogenic variants. HCA was performed using Euclidean distances and the average linkage agglomeration algorithm. Numbers reported below each column in the heatmap correspond to the different samples and are the same reported in PCA score plots and biplots in the main manuscript. Figure S5: Dendrogram with heatmap obtained by Hierarchical Cluster Analysis (HCA) on autoscaled data concerning the intra-class relative abundances of CLs resulting from the HILIC-ESI(−)-HRMS analysis of mitochondrial lipid extracts obtained from Opa1^−/−^ MEFs expressing isoform 1 of wild-type human OPA1 (ISO1) or four of its pathogenic variants. HCA was performed using Euclidean distances and the average linkage agglomeration algorithm. Numbers reported below each column in the heatmap correspond to the different samples and are the same reported in PCA score plots and biplots in the main manuscript.

## Abbreviations

The following abbreviations are used in this manuscript:

AGC	Automatic Gain Control
AIF	All Ion Fragmentation
BMP	Bis-monoacylglycerophosphates
CL	Cardiolipins
DNTB	Dithionitrobenzoic acid
ESI	ElectroSpray Ionization
HCA	Hierarchical Cluster Analysis
HCD	Higher-energy Collisional Dissociation
HESI	Heated Electrospray Ionization
HILIC	Hydrophilic Interaction Liquid Chromatography
IMM	Inner Mitochondrial Membrane
MAM	Mitochondria-Associated Membranes
MEF	Mouse Embryonic Fibroblasts
MFN1/MFN2	Mitofusin 1/Mitofusin 2
mtDNA	Mitochondrial DNA
NCE	Normalized Collisional Energy
NEFA	Non-esterified fatty acids
OMM	Outer Mitochondrial Membrane
OPA1	Optic Atrophy 1
OXPHOS	Oxidative Phosphorilation
PC	Phosphatidylcholines
PCA	Principal Component Analysis
PE	Phosphatidylethanolamines
PG	Phosphatidylglycerols
PI	Phosphatidylinositols
PL	Phospholipids
PS	Phosphatidylserines
RPLC	Reversed Phase Liquid Chromatography
XIC	Extracted Ion Current
