# Supplementary material for: Lipidomics Based on HILIC-ESI-HRMS Reveal Subtle Changes in the Mitochondrial Phospholipidome of Mouse Embryonic Fibroblasts Expressing Pathogenic Variants of Human OPA1 Protein
Source: Molecules. 2026 Aug 5;31(15):2719. doi: 10.3390/molecules31152719 (PMC13468052; doi:10.3390/molecules31152719)
Supplement: Supplementary file 1 [file molecules-31-02719-s001.zip › molecules-4398527-supplementary.pdf]

Supplementary Information

Lipidomics Based on HILIC-ESI-HRMS Reveal Subtle Changes in the Mitochondrial Phospholipidome of Mouse Embryonic Fibroblasts Expressing Pathogenic Variants of Human OPA1 Protein

Andrea Castellaneta [1], Ilario Losito [1,2,*], Vito Porcelli [3], Serena Barile [3], Alessandra Maresca [4], Valentina Del Dotto [5], Penelope Magnoni [4], Claudia Zanna [5], Ludovica Sofia Guadalupi [1], Valeria Cinquepalmi [1], Cosima Damiana Calvano [1,2], David C. Chan [6], Valerio Carelli [4,5], Luigi Palmieri [3,7] and Tommaso R. I. Cataldi [1,2]

[1] Dipartimento di Chimica, Università degli Studi di Bari Aldo Moro, Via Orabona 4, 70126 Bari, Italy
[2] Centro Interdipartimentale di Spettrometria di Massa per Ricerche Tecnologiche (SMART), Università degli Studi di Bari Aldo Moro, Via Orabona 4, 70126 Bari, Italy
[3] Dipartimento di Bioscienze, Biotecnologie e Ambiente, Università degli Studi di Bari Aldo Moro, Via Orabona 4, 70126 Bari, Italy
[4] Programma di Neurogenetica, IRCCS Istituto delle Scienze Neurologiche di Bologna, Via Altura 3, 40139 Bologna, Italy
[5] Dipartimento di Scienze Biomediche e Neuromotorie, Università degli Studi di Bologna, Via Altura 3, 40139 Bologna, Italy
[6] Division of Biology and Biological Engineering, California Institute of Technology, 1200 East California Boulevard, Pasadena, CA 91125, USA
[7] Consiglio Nazionale delle Ricerche (CNR)-Istituto di Biomembrane, Bioenergetica e Biotecnologie Molecolari, Via Giovanni Amendola, 122/O, 70126 Bari, Italy
* Correspondence: ilario.losito@uniba.it; Tel.: +39-080-5442506

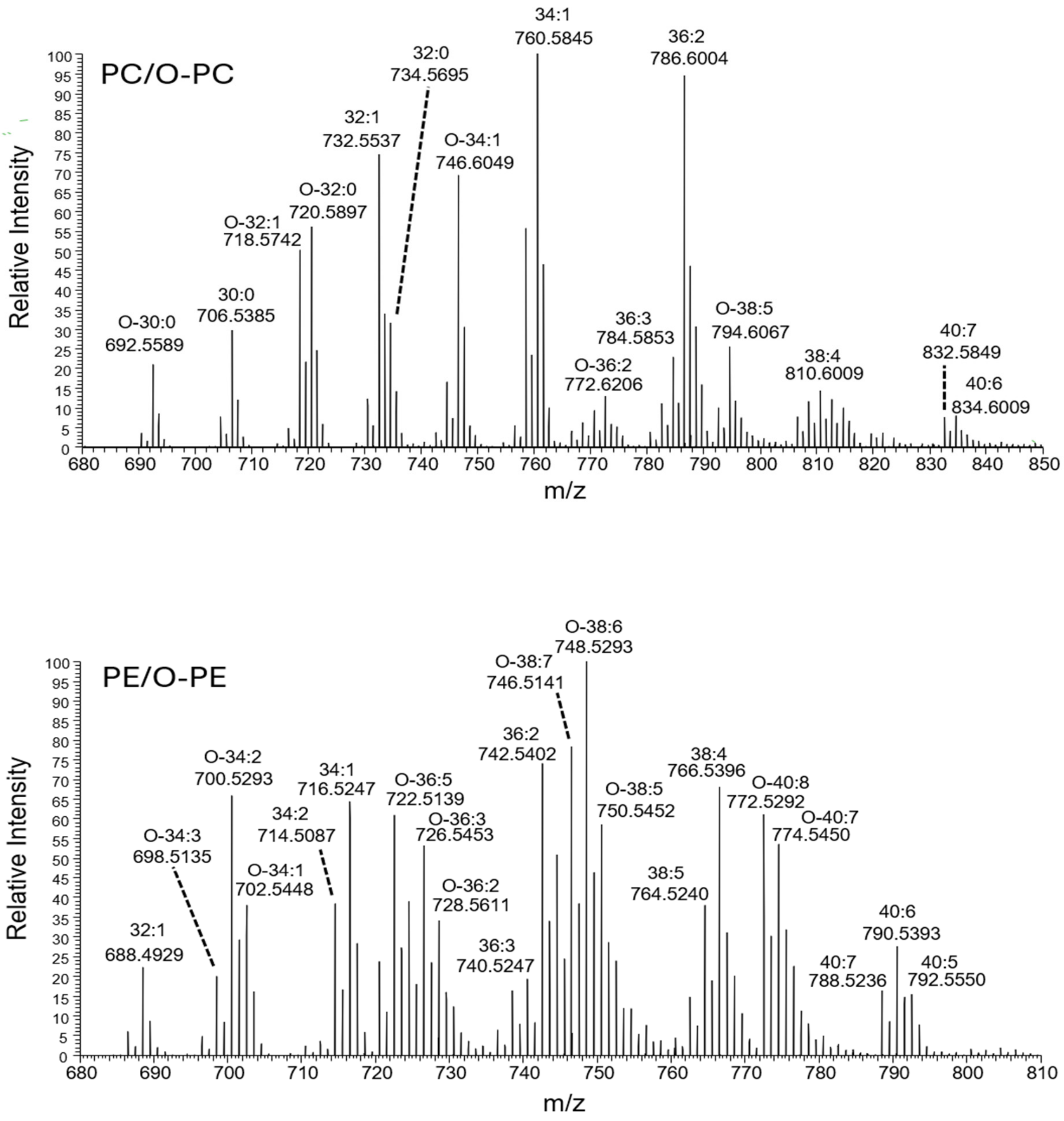

Figure S1. HRMS spectra averaged under chromatographic bands referred to PCs and O-PCs (upper panel), and PEs and O-PEs (lower panel), obtained after the HILIC-ESI-HRMS analysis, in positive and negative polarity, respectively, of the mitochondrial lipid extract referred to a sample of Opa1$^{-/-}$ MEFs expressing isoform 1 of wild-type human OPA1 (ISO1). For the sake of clarity, only peak signals referred to the most abundant monoisotopic ions were labelled with the sum composition of the corresponding PL. In the case of alk(en)yl/acyl PL the sum composition label is reported as O-C:D, with C and D representing the total number of carbon atoms and C=C bonds of the side chains.

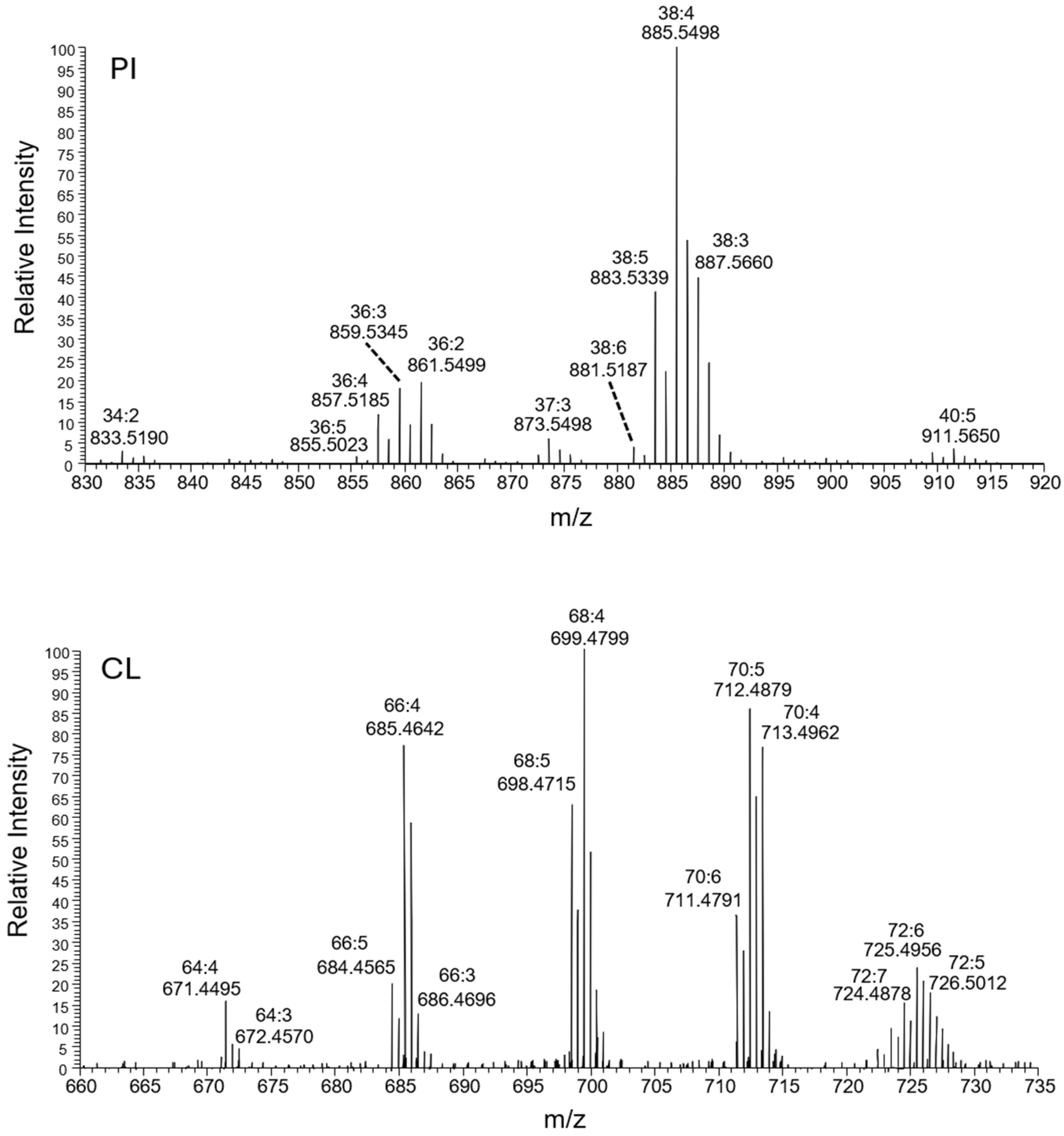

Figure S2. HRMS spectra averaged under chromatographic bands referred to PIs (upper panel) and CLs (lower panel), obtained after the HILIC-ESI-HRMS analysis in negative polarity of the mitochondrial lipid extract referred to Opa1$^{-/-}$ MEFs expressing isoform 1 of wild-type human OPA1 (ISO1). For the sake of clarity, only peak signals referred to the most abundant monoisotopic ions were labelled with the sum composition of the corresponding PL. The PLs sum composition label is reported as C:D, with C and D representing the total number of carbon atoms and C=C bonds of the side chains.

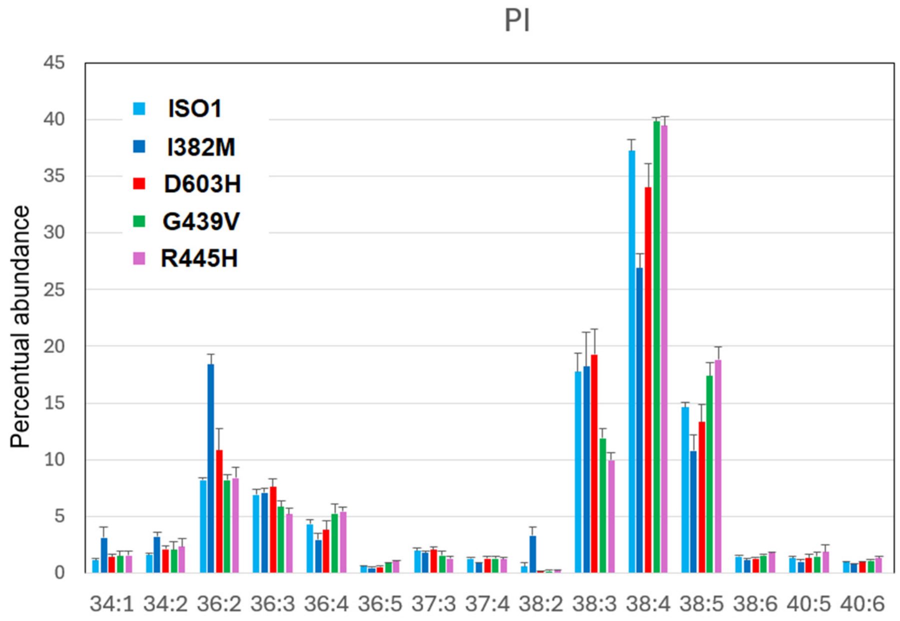

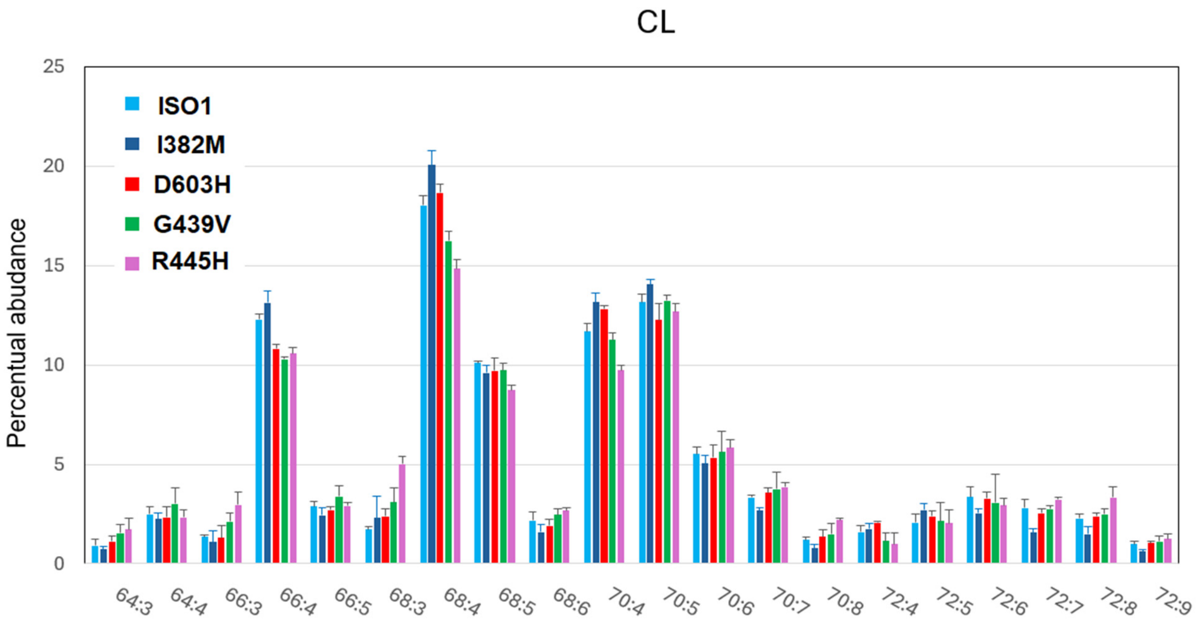

Figure S3. Percentual abundance values related to specific sum compositions recognized for PI and CL classes after the HILIC-ESI(−)-HRMS analysis of mitochondrial lipid extracts obtained from Opa1$^{-/-}$ MEFs expressing isoform 1 of wild-type human OPA1 (ISO1) or four of its pathogenic variants (I382M, D603H, G439V, R445H). Average values obtained for three biological replicates and error bars representing standard deviations are reported.

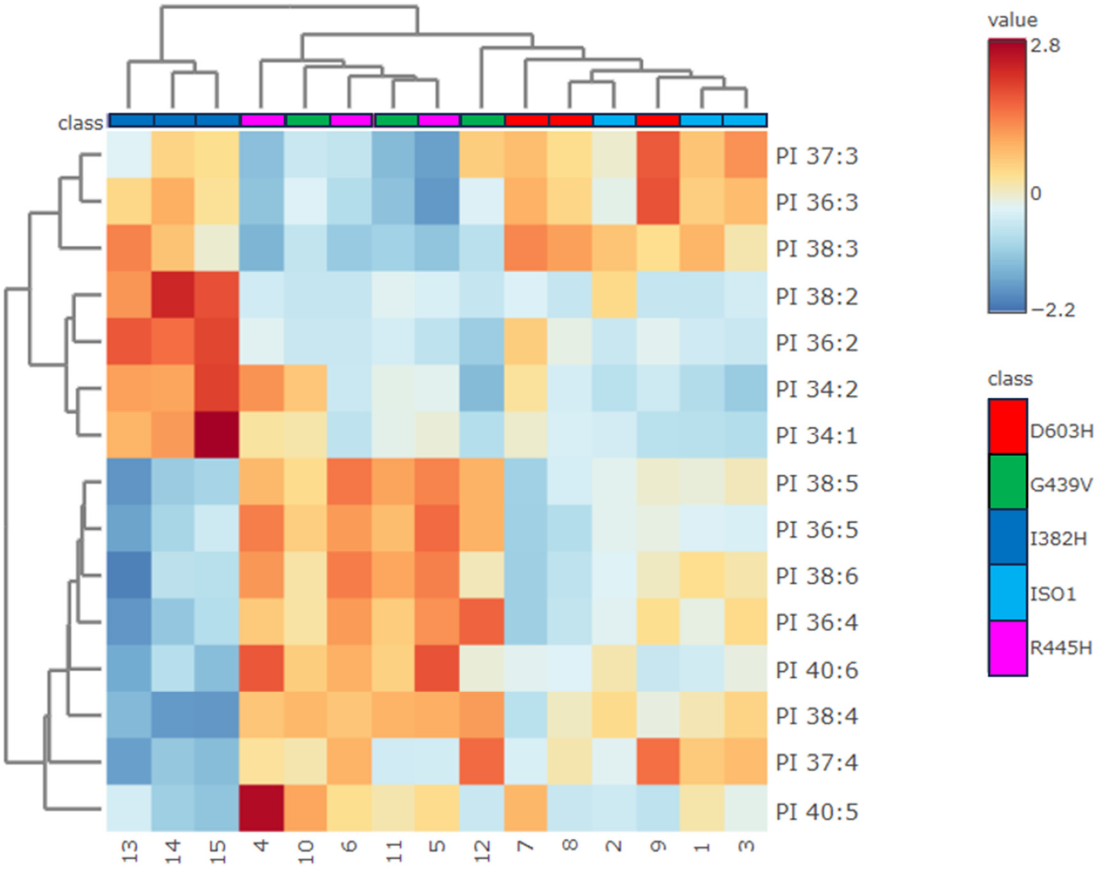

Figure S4. Dendrogram with heatmap obtained by Hierarchical Cluster Analysis (HCA) on autoscaled data concerning the intra-class relative abundances of PIs resulting from the HILIC-ESI(−)-HRMS analysis of mitochondrial lipid extracts obtained from Opa1$^{-/-}$ MEFs expressing isoform 1 of wild-type human OPA1 (ISO1) or four of its pathogenic variants. HCA was performed using Euclidean distances and the average linkage agglomeration algorithm. Numbers reported below each column in the heatmap correspond to the different samples and are the same reported in PCA score plots and biplots in the main manuscript.

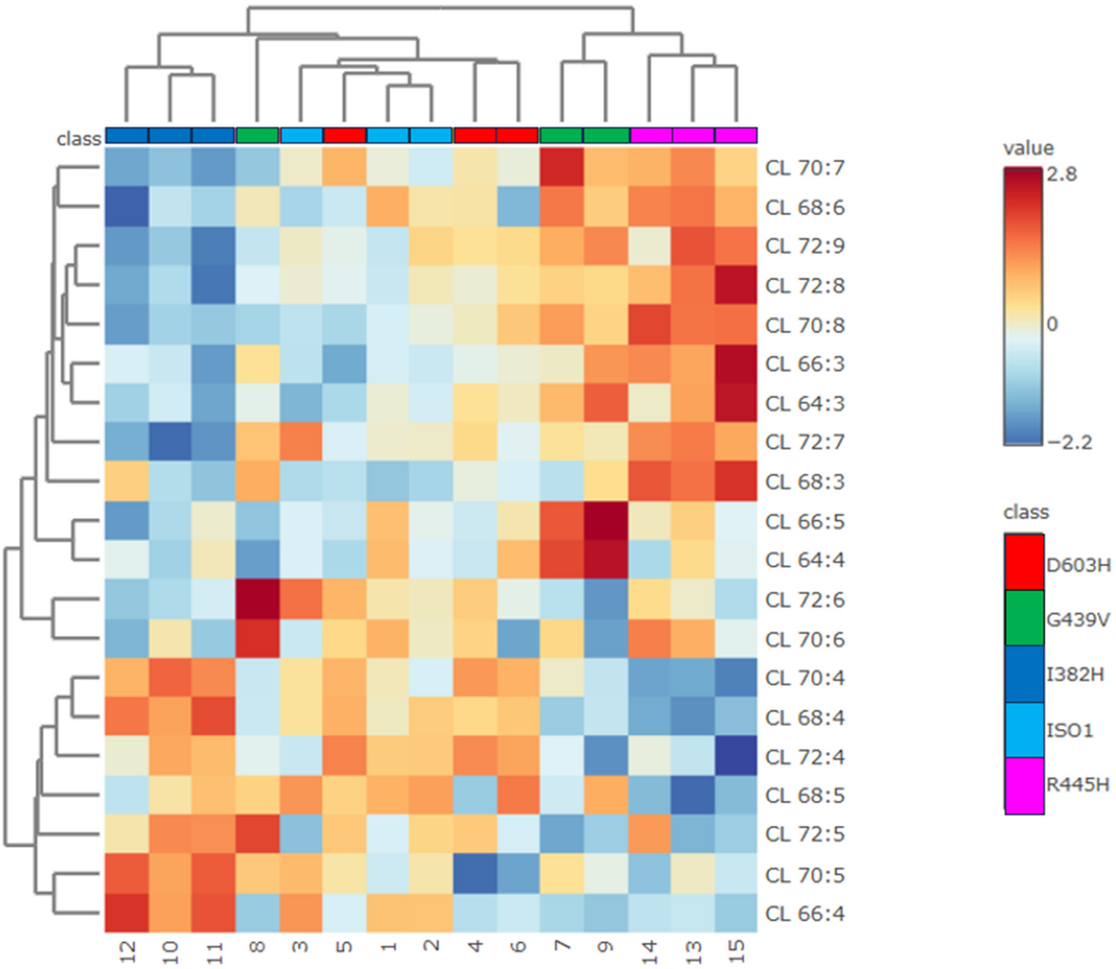

Figure S5. Dendrogram with heatmap obtained by Hierarchical Cluster Analysis (HCA) on autoscaled data concerning the intra-class relative abundances of CLs resulting from the HILIC-ESI(−)-HRMS analysis of mitochondrial lipid extracts obtained from Opa1$^{-/-}$ MEFs expressing isoform 1 of wild-type human OPA1 (ISO1) or four of its pathogenic variants. HCA was performed using Euclidean distances and the average linkage agglomeration algorithm. Numbers reported below each column in the heatmap correspond to the different samples and are the same reported in PCA score plots and biplots in the main manuscript.
